# Supplementary material for: A simplified approach for efficiency analysis of machine learning algorithms
Source: PeerJ Comput Sci. 2024 Nov 28;10:e2418. doi: 10.7717/peerj-cs.2418 (PMC11623197; doi:10.7717/peerj-cs.2418)
Supplement: Supplemental Information 1 [file peerj-cs-10-2418-s001.docx]

**Appendix A**

**Illustration (Figure 1 & Figure 2) of Efficiency Analysis of Machine Learning Algorithms for Medical Image Analysis**

This section provides an in-depth analysis of the efficiency of the machine learning algorithms on the BraTS 2020 dataset for medical image analysis. We used Support Vector Machines (SVMs), Random Forests, and Neural Networks to classify tumor regions in MRI scans, as these algorithms are well-suited for high-dimensional medical data. The features considered for training and prediction included MRI images (T1, T1Gd, T2, and FLAIR) and labeled tumor regions (enhancing tumor, tumor core, and edema). The rationale for choosing these algorithms is their robustness and effectiveness in handling complex data and improving the accuracy of medical diagnoses.

**Collect Raw Metrics**

For the medical image analysis application, we assumed the following raw metrics for some ML algorithms:

Training Time: 100 seconds

Prediction Time: 2 seconds

Memory Usage: 1500 MB

CPU Usage: 60%

GPU Usage: 30%

RAM Usage: 1500 MB

Scalability: Training time increases from 100 s (for 10k images) to 500 s (for 50k images)

Robustness: Accuracy deviation of only 2% under noisy conditions

Adaptability: Minor adjustments needed to handle different image types

Each metric was normalized using the min-max normalization formula, and the composite efficiency score was calculated using the pairwise comparison matrix. The focus of medical image analysis is on prediction time and robustness owing to the critical need for timely and reliable diagnostics​.

#### **Normalize Metrics**

Each metric is normalized using the min-max normalization formula (Equation 1). Figure 3 shows the values of the normalized metrics for medical image analysis. Assume the following min and max values for normalization:

- Training Time: T_min_ = 50 seconds, T_max_​=300 seconds
- Prediction Time: T_min_ = 1 second, T_max_​=10 seconds
- Memory Usage: M_min_ = 1000 MB, M_max_​=5000 MB
- CPU Usage: U_min_ = 10% U_max_ ​= 100%
- GPU Usage: U_min_ = 0% U_max_ ​= 100%
- RAM Usage: U_min_ = 1000 MB U_max_ ​= 5000 MB
- Scalability: S_min_ = 1 (ideal scaling) and S_max_ = 10 (poor scaling)
- Robustness: R_min_ = 0.5 (ideal robustness); R_max_ = 10 (poor robustness)
- Adaptability: A_min_ = 0.5 (ideal adaptability); A_max_ = 10 (poor adaptability)


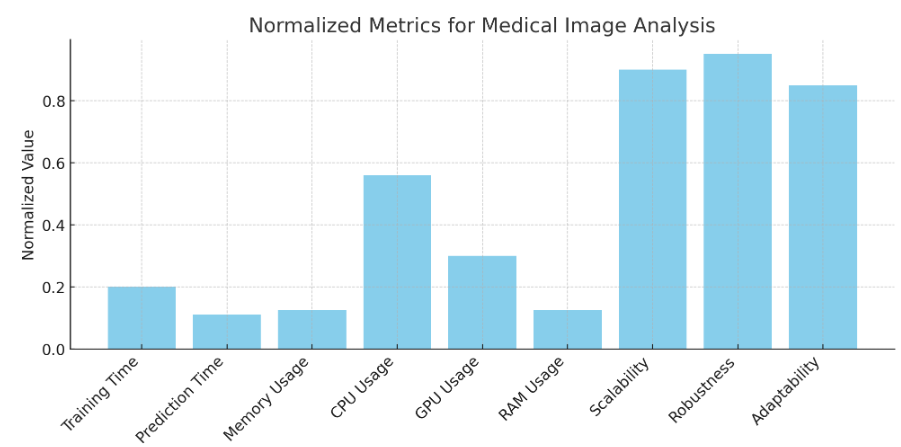


Figure 3. Normalized Metrics for Medical Image Analysis

**Create Pairwise Comparison Matrix**

Using AHP, a pairwise comparison matrix was created for the metrics based on their relative importance in medical image analysis applications. For instance, prediction time and robustness might be prioritized because of the need for quick and reliable diagnostics. Figure 4 shows the pairwise comparison matrix for medical image analysis.

#### **
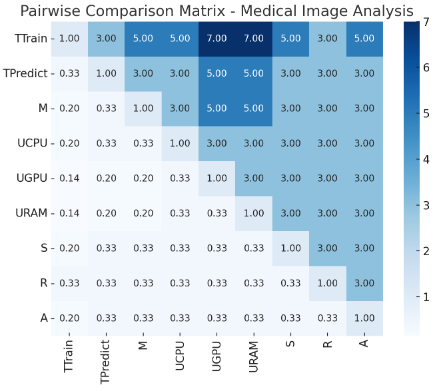
**

Figure 4. Pair wise Comparison Matrix for Medical Image Analysis

#### **Calculate Normalized Weights**

1. **Sum each column**: Calculate the sum of each column in the pairwise comparison matrix.
2. **Normalize the matrix**: Divide each element by the sum of its respective column.
3. **Calculate the average of each row** is calculated, and the average of the normalized values in each row gives the normalized weight for each metric.

**Normalized Weights for Medical Image Analysis:**

- Training Time: $w_{1}$= 0.124
- Prediction Time: $w_{2}$=0.182
- Memory Usage: $w_{3}$=0.089
- CPU Usage: $w_{4}$=0.068
- GPU Usage: $w_{5}$=0.049
- RAM Usage: $w_{6}$=0.049
- Scalability: $w_{7}$=0.089
- Robustness: $w_{8}$=0.182
- Adaptability: $w_{9}$=0.168

#### **Calculating Composite Efficiency Score for ML Algorithm of Medical Image Analysis:**

The normalized metrics and their corresponding weights are substituted into Equation (2) to calculate the composite efficiency score for medical image analysis. Figure 6 depicts the composite efficiency score for the medical image analysis.

Normalized Metrics (assumed for illustration purposes):

- N( $T_{\mathrm{Train}}$)=0.2
- N($T_{\mathrm{Predict}}$)=0.11
- N(M)=0.125
- N($U_{\mathrm{CPU}}$)=0.56
- N($U_{\mathrm{GPU}}$)=0.3
- N($U_{\mathrm{RAM}}$)=0.125
- N(S)=0.9
- N(R)=0.95
- N(A)=0.85

**Composite Efficiency Score:**

E=0.124⋅0.2+0.182⋅0.11+0.089⋅0.125+0.068⋅0.56+0.049⋅0.3+0.049⋅0.125+0.089⋅0.9+0.182⋅0.95+0.168⋅0.85

E=0.0248+0.02002+0.011125+0.03808+0.0147+0.006125+0.0801+0.1729+0.1428 ≈ **0.51075**


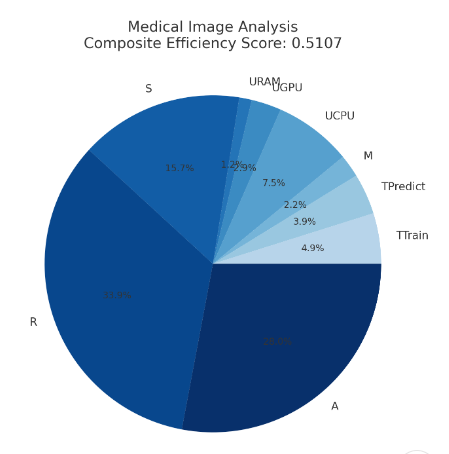


Figure 5. Composite Efficiency Score for Medical Image Analysis
