# Supplementary material for: A simplified approach for efficiency analysis of machine learning algorithms
Source: PeerJ Comput Sci. 2024 Nov 28;10:e2418. doi: 10.7717/peerj-cs.2418 (PMC11623197; doi:10.7717/peerj-cs.2418)
Supplement: Supplemental Information 2 [file peerj-cs-10-2418-s002.docx]

**Appendix B**

**Illustration (Figure 1 & Figure 2) of Efficiency Analysis of Machine Learning Algorithms - Agriculture Dataset for Crop Prediction**

#### This section analyzes the efficiency of the machine learning algorithms on the Kaggle crop-recommendation dataset. The features considered included environmental factors such as nitrogen (N), phosphorus (P), potassium (K), temperature, humidity, pH, and rainfall. We applied Random Forests, k-nearest neighbors (k-NN), and Neural Networks to predict optimal crops based on soil and environmental data because these algorithms are known for their scalability and adaptability in agricultural applications.

#### **Collect Raw Metrics**

For the agricultural data analysis application, assume we have the following raw metrics:

- Training Time: 200 seconds
- Prediction Time: 5 seconds
- Memory Usage: 2500 MB
- CPU Usage: 80%
- GPU Usage: 50%
- RAM Usage: 2500 MB
- **Scalability: Training time increases from 200 s (for 100k records) to 800 s (for 500k records)**
- **Robustness: Accuracy deviation of 3% under noisy and imbalanced data**
- **Adaptability: Moderate adjustments needed to handle new data types**

#### **The composite efficiency score was calculated similarly, emphasizing scalability and adaptability, which are crucial for managing diverse and large-scale agricultural datasets.**

#### **Normalize Metrics**

Each metric is normalized using the min-max normalization formula (Equation 1). Assume the same min and max values for normalization as in the medical image analysis application. The normalized values are presented in Figure 7.


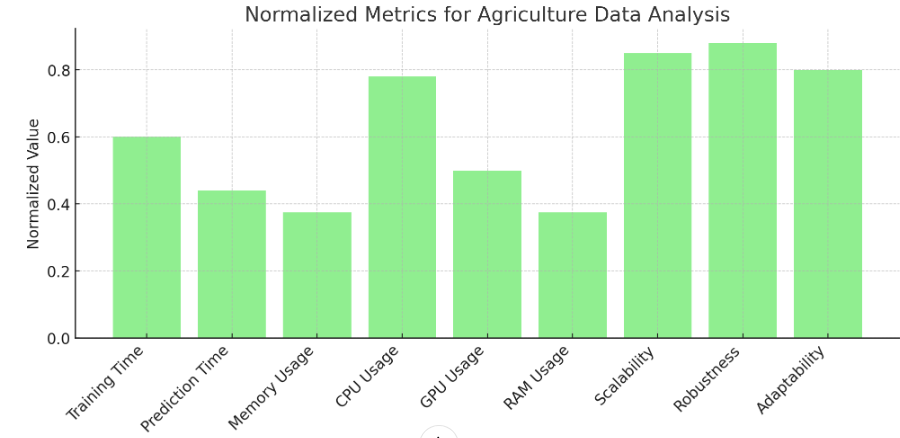


Figure 6. Normalized Metrics for Agriculture Data Analysis

#### **Create Pairwise Comparison Matrix**

Using AHP, a pairwise comparison matrix is created for the metrics based on their relative importance in the agricultural data analysis application. For instance, scalability and adaptability might be prioritized because of the need to handle varying data sizes and types. The pairwise comparison matrix for the agricultural data analysis is shown in Figure 8.

**
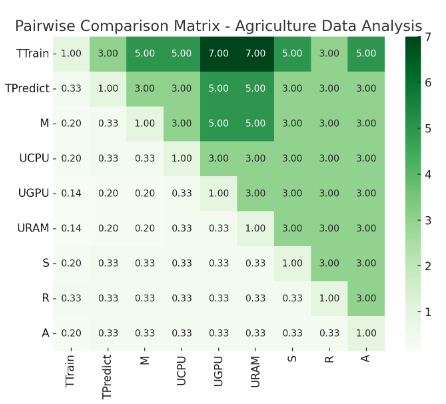
**

Figure 7. Pair wise Comparison Matrix – Agriculture Data Analysis

**Normalized Weights for Agricultural Data Analysis:**

- Training Time: $w_{1}$= 0.113
- Prediction Time: $w_{2}$=0.178
- Memory Usage: $w_{3}$=0.078
- CPU Usage: $w_{4}$=0.062
- GPU Usage: $w_{5}$=0.053
- RAM Usage: $w_{6}$=0.053
- Scalability: $w_{7}$=0.117
- Robustness: $w_{8}$=0.162
- Adaptability: $w_{9}$=0.182

#### **Calculating Composite Efficiency Score for Agricultural Data Analysis**

The normalized metrics and their corresponding weights were substituted into equation (2) to calculate the composite efficiency score for agricultural data analysis. Normalized Metrics (assumed for illustration purposes)

- N( $T_{\mathrm{Train}}$)=0.6
- N($T_{\mathrm{Predict}}$)=0.44
- N(M)=0.375
- N($U_{\mathrm{CPU}}$)=0.78
- N($U_{\mathrm{GPU}}$)=0.5
- N($U_{\mathrm{RAM}}$)=0.375
- N(S)=0.85
- N(R)=0.88
- N(A)=0.8

**Composite Efficiency Score:**

E=0.113⋅0.6+0.178⋅0.44+0.078⋅0.375+0.062⋅0.78+0.053⋅0.5+0.053⋅0.375+0.117⋅0.85+0.162⋅0.88+0.182⋅0.8

E=0.0678+0.07832+0.02925+0.04836+0.0265+0.019875+0.09945+0.14256+0.1456 ≈ **0.6577** .

The composite efficiency score of agriculture data analysis is shown in Figure 9.


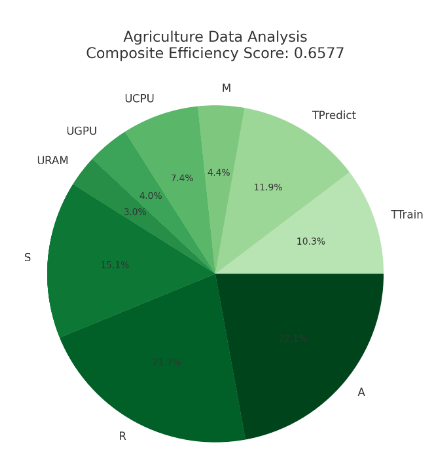


Figure 8. Composite Efficiency Score of Agriculture Data Analysis
